# Impact of Tomosynthesis Acquisition on 3D Segmentations of Breast Outline and Adipose/Dense Tissue with AI: A Simulation-Based Study

**DOI:** 10.3390/tomography9040103

**Published:** 2023-07-03

**Authors:** Bruno Barufaldi, Jordy Gomes, Thais G. do Rego, Yuri Malheiros, Telmo M. Silva Filho, Lucas R. Borges, Raymond J. Acciavatti, Suleman Surti, Andrew D. A. Maidment

**Affiliations:** 1 Department of Radiology, University of Pennsylvania, Philadelphia, PA 19104, USA; racci@pennmedicine.upenn.edu (R.J.A.); surti@pennmedicine.upenn.edu (S.S.); andrew.maidment@pennmedicine.upenn.edu (A.D.A.M.); 2 Center of Informatics, Federal University of Paraiba, Joao Pessoa 58051-900, PB, Brazil; jordyvieira11@gmail.com (J.G.); thais@ci.ufpb.br (T.G.d.R.); yuri@ci.ufpb.br (Y.M.); 3 Department of Engineering Mathematics, University of Bristol, Bristol BS8 1QU, UK; telmo.silvafilho@bristol.ac.uk; 4 Real Time Tomography, LCC, Villanova, PA 19085-1801, USA; lucas.borges@realtimetomography.com

**Keywords:** multi-modality imaging, digital breast tomosynthesis, breast cancer, screening, virtual clinical trials, artificial intelligence

## Abstract

Digital breast tomosynthesis (DBT) reconstructions introduce out-of-plane artifacts and false-tissue boundaries impacting the dense/adipose and breast outline (convex hull) segmentations. A virtual clinical trial method was proposed to segment both the breast tissues and the breast outline in DBT reconstructions. The DBT images of a representative population were simulated using three acquisition geometries: a left–right scan (conventional, I), a two-directional scan in the shape of a “T” (II), and an extra-wide range (XWR, III) left–right scan at a six-times higher dose than I. The nnU-Net was modified including two losses for segmentation: (1) tissues and (2) breast outline. The impact of loss (1) and the combination of loss (1) and (2) was evaluated using models trained with data simulating geometry I. The impact of the geometry was evaluated using the combined loss (1&2). The loss (1&2) improved the convex hull estimates, resolving 22.2% of the false classification of air voxels. Geometry II was superior to I and III, resolving 99.1% and 96.8% of the false classification of air voxels. Geometry III (Dice = (0.98, 0.94)) was superior to I (0.92, 0.78) and II (0.93, 0.74) for the tissue segmentation (adipose, dense, respectively). Thus, the loss (1&2) provided better segmentation, and geometries T and XWR improved the dense/adipose and breast outline segmentations relative to the conventional scan.

## 1. Introduction

Digital breast tomosynthesis (DBT) is a screening imaging modality with increased cancer detection and reduced recall rates compared with its predecessor, digital mammography (DM) [1,2,3]. Despite substantial advantages over DM [2,4], DBT still lacks prognostic capability when compared to magnetic resonance, contrast-enhanced mammography, or molecular imaging [5,6,7,8,9,10]. Thus, alternative geometries for DBT have been widely investigated by our group to address the tomosynthesis limitations [11,12,13,14].

Our lab at the University of Pennsylvania is developing a next-generation tomosynthesis (NGT) prototype capable of multi-modality imaging with positron emission tomography (PET) and DBT [15,16]. The DBT acquisition incorporates an additional degree of freedom in the scanning motion not used in current clinical systems; specifically, posteroanterior (PA) scanning perpendicular to the chest wall. The ultimate goal is to co-register anatomic information in DBT with radiotracer uptake in PET. To localize the radiotracer signal with the breast anatomy, it is necessary for there to be precise segmentations of the dense/adipose tissue as well as the 3D breast outline in the DBT reconstruction.

In previous studies, we developed a virtual clinical trial (VCT) method to evaluate the accuracy of attenuation correction with DBT for the PET detector used in our dedicated prototype [15,16]. Using VCTs, we demonstrated that DBT can facilitate the attenuation correction of breast tissue for functional breast imaging, potentially improving the reconstruction of coincident gamma rays [15]. The volume, size, and breast tissue estimates can be improved using our proposed multi-modality (PET–DBT) prototype [15,16]. Our prototype has been designed, developed, and validated using computer simulations of breasts and test objects scanned in the NGT system [12,16,17,18].

Our computer simulation-based methods have been incorporated into an OpenVCT framework [19,20]. The OpenVCT framework allows the simulation of the curvature of the breast under mammography compression [21,22]. Our previous work has shown that breasts under compression result in “false boundaries” of the regions near the breast and air (i.e., inaccuracies in the 3D breast outline segmentation or “convex hull”) in DBT reconstructions [12,15,23]. The convex hull effect can be better visualized in DBT images that are reconstructed using simple backprojection algorithms; the combination of the X-ray attenuation of air and tissues contributes to the backprojection sum of the signal in the convex hull.

Rodríguez-Ruiz et al. have developed a principal component analysis (PCA) method to characterize and model the compressed breast curvature between the support table and the compression paddle [21,22]. This method has been used to create various patient-based 3D models, enabling the accurate estimate of the convex hull area of breasts under mammography compression [24]. Accurate segmentation of the breast curvature is important to improve image processing and/or reconstruction in regions near to the breast skin [21]. Importantly, the ground truth of the 3D curvature cannot be obtained in patient images due to limitations in the acquisition geometry of clinical DBT systems [25,26].

Clinical DBT systems use a limited angle acquisition with a small number of low-dose projections that can result in out-of-plane artifacts of breast tissue in the DBT reconstructions [24,27]. The accurate estimate of breast density using DBT images is challenging because of the presence of out-of-plane artifacts and the superposition of breast tissue [28]. Fully automated systems have been developed to quantify fibroglandular tissue in DBT images [29,30]. The methods used to quantify breast density typically rely on physics-based models [31]. These models are usually derived from physical phantoms, which may present extrapolation methods of density based on the compressed breast thickness. Extrapolations of breast density from physics-based models may not be appropriate for women from every imaging center [31].

VCTs have been widely used to model patient-specific breasts, design imaging systems, and simulate human interpretation [20,32,33,34]. Today, the use of VCTs in breast imaging is recommended and supported by the U.S. Food and Drug Administration to design new medical systems [35]. In this study, we proposed a U-Net- and VCT-based method to optimize the NGT acquisitions to: (1) reduce the superposition of dense and adipose tissue in DBT reconstructions and, simultaneously, (2) segment the convex hull. The breasts were simulated using the data distribution of women enrolled in a breast screening program [31]. A nnU-Net was modified and trained using input data simulated using three DBT acquisition geometries of the NGT system. The results obtained from the nnU-Net were compared with actual ground-truth to evaluate the changes in performance between the segmentation models.

## 2. Materials and Methods

### 2.1. Breast Outline Simulation

**Figure 1:**
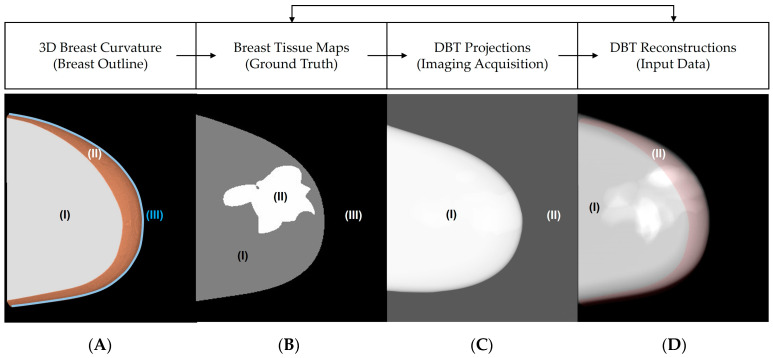
(**A**) Example of breast outline under mammography compression: (**A.I**) area in contact with the compression paddle, (**A.II**) convex hull area, and (**A.III**) area in contact with the breast support. (**B**) Central slice of breast phantom: (**B.I**) adipose tissue, (**B.II**) dense tissue, and (**B.III**) air. (**C**) Central DBT projection: (**C.I**) breast, and (**C.II**) background (air). (**D**) Central slice of DBT reconstruction (raw data): ((**D.I**), shaded in red) area least, and (**D.II**) most affected by the convex hull.

The breast outlines were simulated using PCA-based software that models breasts under mammographic compression [21,22]. The PCA software models the compressed breast curvature and accurately characterizes the compression force applied between the breast support and the compression paddle. The compressed breast thickness (CBT) of the outlines was simulated following the clinical data distribution of representative patients enrolled in the screening program from the Hospital of the University of Pennsylvania (Table 1) [2,31]. In total, 550 outlines of 0.1 mm^3^ voxel size were modeled under craniocaudal (CC) compression (Figure 1).

The PCA software can simulate realistically the convex hull volume resulting from the top and bottom surfaces of the breast delineated by the compression paddle and the breast support. Figure 1A illustrates an example of the 3D breast curvature simulated using the PCA software. Note that this software simulates the breast (highlighted in yellow) and air gaps (Figure 1.II) between the compression paddle and breast support commonly missed in DBT reconstructions.

### 2.2. Breast Density Simulation

The internal composition of the breast tissue was modeled using an octree-based software developed and incorporated into the OpenVCT framework [19,36]. The percent of volumetric breast density (%VBD) was simulated and matched with the clinical data distribution of screening patients (Table 1) [31]. The breast anatomies were simulated with the mean VBD = 13.59 (9.00, 20.60)% and mean CBT = 56.10 (49.05, 66.65) mm. In this work, Cooper’s ligament, breast compartments, lesions, and additional fine breast structures were not simulated to simplify the complexity of the breast anatomies and to categorize the ground truth into three classes: air, adipose, and dense tissue (phantom mask, Figure 1B) [15,16].

The conventional geometry (Table 2.I) encompassed 15 projections with source motion in the left–right (LR) direction. The “T” geometry (Table 2.II) incorporated seven projections with source motion in the PA direction and eight projections with source motion in the LR direction (i.e., LR) [28]. Finally, we simulated a hypothetical acquisition geometry (Table 2.III, extra-wide angular range or XWR), since previous work had demonstrated that wide-range scanning offers the most accurate segmentation of the 3D breast outline or “convex hull” [24]. The XWR geometry included a total of 91 projections over a wide (90°) angular range in the LR direction with a radius of 738 mm and the pivot point at the breast support and 6-times higher dose than the conventional or T geometry. Although the scanning range is broader and the dose is larger than the current clinical DBT systems, the XWR geometry is useful for the purpose of a thought experiment in dense/adipose and breast outline segmentations.

The exposure settings were simulated using the automatic exposure control (AEC) data for a system with 15 projections [15]. For the XWR geometry, the increased number of projections (91) resulted in a 6-fold increase in the dose (6-fold higher mAs at the same kV and target/filter combination). The attenuation coefficient data of the materials used to simulate the phantoms came from the ICRU Report 44 [37]. Reconstructed DBT slices (0.085 mm isotropic voxel dimensions) were produced using simple backprojection without any image filtering (Briona, version 7.12, Real-Time Tomography, Villanova, PA, USA) [38].

The phantom masks (0.100 mm^3^) were zero-padded and matched to the voxel resolution of the reconstructed slices (0.085 mm^3^). Both the phantom masks and reconstructed slices were used as input volumes for training the nnU-Net. The input volumes were down-sampled to approximately 20% of the original dimensions resulting in voxel sizes of 551 × 701 × *T*, where *T* was the thickness of each phantom. The aspect ratio of the detector was maintained (width-to-height = 0.79).

### 2.3. Convex Hull and Tissue Segmentations

The full-resolution 3D U-Net implementation from the nnU-net framework [39] was used to segment the simulated breast tissue. The nnU-net framework has been successfully used for the 3D segmentation of biomedical images [39]. This framework is considered efficient for volume segmentation, especially for anisotropic volumes; embedded pre-processing methods for estimating bounding boxes and cropping (zero values) of the object are used to reduce the amount of allocated CPU and GPU memory and to accelerate the training process.

In this study, we proposed a multi-task multi-class segmentation method for training the nnU-Net. The models were trained for segmentation using the focal Tversky loss (FTL) [40,41]. Two loss functions were calculated: (1) FTL for the multi-class segmentation of adipose and dense tissue, and (2) FTL for binary segmentation of air and tissue. FTL (1) was used to maximize the differentiation between the adipose and dense tissues. FTL (2) was used to minimize the FP and FN segmentation errors calculated of the convex hull. Both FTLs were derived from the original Tversky index ($TI_{c}$):

$$TI_{c}=\frac{\sum_{i=1}^{N}p_{ic}g_{ic}+\varepsilon }{\sum_{i=1}^{N}p_{ic}g_{ic}+\alpha \sum_{i=1}^{N}p_{i\bar{c}}g_{ic}+\beta \sum_{i=1}^{N}p_{ic}g_{i\bar{c}}+\varepsilon }$$

$$FTL_{c}=\sum_{c}^{}{\left(1-TI_{c}\right)}^{\gamma },$$

where $p_{ic}$ and $p_{i\bar{c}}$ are the probability of a pixel *i* being in a positive (*c*) class or negative ($\bar{c}$) class. The terms $g_{ic}$ and $g_{i\bar{c}}$ represent the ground-truth classes ($g_{ic}\;\mathrm{and}\;g_{i\bar{c}}\in \left\{0,1,2\right\}$). *N* is the total number of pixels in the image. The term ε provides numerical stability to prevent division by zero [41]. The FTL has a better tradeoff between the precision and recall rates when training, addressing the data imbalance in image segmentation. The α, *β*, and *γ* hyperparameters were determined based on prior work (*α* = 0.7, *β* = 0.3, and *γ* = 3/4) [39].

Our proposed method for FTL (1&2) used loss (1) combined with the separate calculation of loss (2) to maximize the accuracy in dense/adipose and breast outline segmentations. Four models were developed to evaluate the changes in performance of the nnU-Net segmentation: model I_1_ was trained using the loss method (1) with the dataset simulated using geometry I; models I_1&2_, II_1&2_, and III_1&2_ were trained using a combined loss (1) and (2) with datasets simulated using geometries I, II, and III, respectively. From this point on in the paper, we adopted the terms I_1_, I_1&2_, II_1&2_, and III_1&2_ to reference the four nnU-Net models developed in this study. The impact of the loss methods was evaluated using the results obtained by models I_1_ and I_1&2_. The impact of acquisition geometry was evaluated using the results obtained by models I_1&2_, II_1&2_, and III_1&2_.

To avoid overfitting in the training process, the nnU-net models were saved every five epochs (batch size = 2); loss and dice metrics were monitored during the training and validation of the nnU-net models. We used the Adam optimizer with a learning rate of 0.01 (default) for training. The models were developed on a workstation equipped with a CPU Intel i5 12,600 k, GeForce RTX 3080 (10 Gb VRAM), and 32 Gb RAM 3600 MHz.

### 2.4. Convex Hull and Tissue Analyses

Our previous study showed that the segmented breast volume is overestimated in the conventional geometry with LR source motion [23]. In this study, the area of the segmented breast was calculated slice-wise and the error in the area of each slice was analyzed as a function of distance (*d*) relative to the central slice. Our previous work showed that slices closer to the central slice (i.e., *d ≈* 0 mm) are characterized by the smallest errors in the breast area estimates. To account for breasts of different thickness, both the convex hull and tissue predictions were binned into 10 distance categories regardless of the phantom thickness.

The mean Jaccard, Dice, precision, recall, and accuracy were calculated for the segmentation models. For convex hull calculations, mean Jaccard distance or Tanimoto index (i.e., ratio of the disjunct breast volume normalized by the union breast volume of ground-truth and prediction) were calculated to report dissimilarities in the breast curvature. We also reported changes in the performance of the convex hull estimates resulting from the four models. Non-parametric Kruskal–Wallis tests by rank and Wilcoxon–Mann–Whitney tests were used to estimate the differences in the means of the data distributions and to analyze significant differences using pairwise comparisons.

Finally, colormaps representing the accuracy of the multi-class predictions were calculated to illustrate the predictions of each model; true positives (TP), true negatives (TN), false positives (FP), and false negatives (FN) were shown.

## 3. Results

The nnU-Net was trained using input volumes, but the results were shown and interpreted by slice as a function of *d*. This allowed us to better understand the models’ predictions in specific regions such as in the segmented breast area.

**Figure 2:**
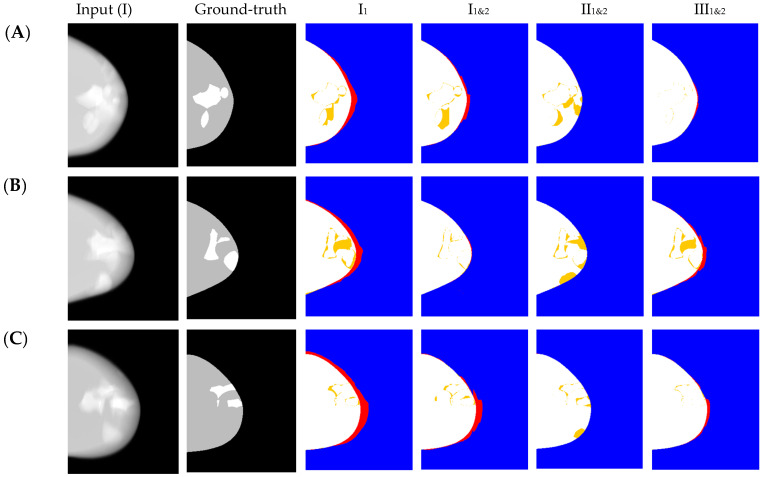
(Left to right) Examples of DBT reconstructions (of conventional NGT geometry) with the most-bottom slice on focus (i.e., in contact with breast support), ground-truth mask (colormap: black = background, gray = adipose tissue, and white = dense tissue), and accuracy maps (colormap: blue = TN background, red = FP or FN background, yellow = FP or FN dense tissue) of models I_1_, I_1&2_, II_1&2_, and III_1&2_. Samples of large phantoms with CBT and CND (**A**) 75 mm and 130 mm, (**B**) 81 mm and 140 mm, and (**C**) 81 mm and 150 mm, respectively.

Model I_1_ overestimated predictions of the adipose tissue for the regions most affected by inaccuracies in the convex hull, resulting in higher number of FPs in these regions (Figure 2, I_1_, highlighted in red). The combined loss (FTL (1&2)) substantially reduced the number of FPs of adipose tissue (Figure 2, I_1&2_) as compared with the predictions of I_1_. T was the superior geometry to estimate the convex hull regions, but it overestimated the predictions of dense tissue (Figure 2, II_1&2_). The XWR geometry with increased number of projections was superior to the conventional or T for segmentation of dense tissue, but still inferior to T for the segmentation of the convex hull (Figure 2, III_1&2_).

The performance between each model to segment the convex hull regions can be better visualized considering the mean Jaccard distance of air categorized by *d* (Table 3). Higher differences in mean Jaccard distances were observed for the extreme ranges of *d* as compared to the central slices. Overall, FTL (1&2) resulted in a 22.2% reduction of misclassification of air voxels. This loss combined with the dataset simulated with the T geometry (model II_1&2_) resulted in the lowest values of Jaccard distance as compared to models I_1_, I_1&2_, and III_1&2_ (*p* < 0.001). The performance of model II_1&2_ represented an improvement in 99.1% (I_1&2_) and 96.8% (III_1&2_) of air voxels misclassified in the convex hull.

The performance of each model for segmentation of (A) adipose and (B) dense tissues is shown in Table 4. For adipose tissue segmentation, model II_1&2_ was superior to I_1_ and I_1&2_; the difference in the means of the Jaccard indices were statistically significant with *p* < 0.001, except for areas in the central-bottom plane of the reconstructions with *d* intervals of [−16.45, −11), [−11, −5.5), and [−5.5, −0.05) mm with *p* = (0.21, 0.51, 0.16). For dense tissue segmentation, model II_1&2_ was inferior to models I_1_ and I_1&2_; the difference in the means of the Jaccard indices were statistically significant with *p* < 0.001, except for the areas in the central and top planes of the reconstructions with *d* intervals of [−5.5, −0.05) mm and >22.3 mm with *p* = (0.087, 0.070). Model III_1&2_ performed the most accurate segmentation for both adipose and dense tissues as compared to the other models (*p <* 0.001).

Model III_1&2_ was superior to the others for segmentation of breast tissue (Table 5). Model II_1&2_ offered the second-best performance for adipose tissue segmentation, but it decreased the overall accuracy of dense tissue segmentation (Table 5B). Still, the Dice and Jaccard coefficients showed similar results in the dense tissue segmentation as compared to model I_1_.

## 4. Discussion

Clinical DBT systems acquire images using source motion with a limited angular range and a limited number of projections, resulting in under-sampled reconstructions with out-of-plane artifacts that complicate the accurate segmentation of breast tissue. Additionally, the segmented breast volume is overestimated in DBT reconstructions; the conventional scanning motion results in increased false-positive tissue artifacts (i.e., false boundaries) in regions adjacent to the actual breast outline. In this study, we investigated the feasibility of varying the number of projections and the direction of the source positions as compared to a hypothetical acquisition geometry (XWR, Table 2.III). Changes in performance between acquisition geometries were evaluated using the volumetric segmentation of simulated breast tissue with nnU-Net.

The default loss function used In the nnU-Net architecture was modified to address the imbalanced class data in DBT reconstructions. Our previous study [42] has shown that FTL results in better segmentation of adipose and dense tissue than traditional cross-entropy loss; the recall and precision rates are improved when a trade-off between segmentation errors (i.e., FP and FN) of breast tissue is considered for training a nnU-Net. In this work, the FTL was used to optimize (simultaneously) the segmentation of the 3D breast outline (convex hull) and the segmentation of dense and adipose tissues from the total (resized) reconstructed DBT volume.

Incorporating the FTL for the binary segmentation of air and tissue significantly improved the accuracy of the segmented breast area per slice. The proposed loss method showed that model I_1&2_ was superior to model I_1_, resulting in lower Jaccard distances (i.e., the disjoint intersection between the air and breast areas) at the topmost (*d* ≥ 5.4 mm) and bottommost (*d* < −11.0 mm) slices from the DBT reconstructions. The segmentation of the convex hull resulted in a reduction of misclassified air voxels of 22.2% (*p* < 0.001).

The T geometry demonstrated promise in terms of improving the accuracy of the 3D breast outline segmentation as compared with conventional LR scanning. When comparing the performance of model II_1&2_ (T) with model I_1&2_ (conventional) or model III_1&2_ (XWR), there was a reduction of misclassified air voxels of 99.1% (*p* < 0.001) and 96.8% (*p* < 0.001), respectively. These results are consistent with our previous study which showed that the use of PA scanning motion in the T geometry does indeed improve the accuracy of the 3D breast outline segmentation, particularly in a scan with a narrow range of LR source motion [24].

This paper also demonstrated that an increased number of projections acquired with an extra-wide scanning range improved the tissue segmentation of the DBT reconstructions. Model III_1&2_ provided the most accurate segmentation of both the adipose and dense tissues. Thus, the advantage of a wide-range scan acquired with increased projections is minimizing the out-of-plane artifacts and improving tissue differentiation. However, the disadvantage is the potential for truncation artifacts, particularly in large breasts. These artifacts arise due to the loss of tissue coverage in the most oblique projections based on the size limitations of the detector field-of-view [26,43].

For the segmentation of adipose tissue, the T geometry presented the second-best DBT reconstructions (II_1&2_). For the segmentation of dense tissue, the conventional geometry presented the second-best DBT reconstructions (I_1&2_). We hypothesize that source motion can introduce additional out-of-plane artifacts in the PA direction, complicating the segmentation of breast tissues in the raw projections. Additional scanning motions and strategies with rapid imaging processing in the projections will be explored in future work to maximize the benefits of the NGT system design and to reduce out-of-plane artifacts in multiple directions of X-ray source motion.

Image acquisition, processing, and reconstruction techniques can be personalized and would potentially benefit from customized scanning motions; advanced and custom-tailored reconstruction techniques can be developed to improve the image quality with reduced out-of-plane artifacts in DBT. Our previous work has demonstrated the benefits of customized acquisitions for: improved visibility of finer and small structures in mastectomy images [28], reduced cone beam artifacts with a superior breast outline demarcation [12,17,44], and improved fibroglandular–adipose tissue contrast with reduced out-of-plane artifacts [15,16]. Our colleagues’ work [21] also emphasized the additional clinical benefits with more accurate information of breast tissue and the convex hull such as improved volumetric tissue estimation and registration [45,46,47] and improved models for 3D printing of phantoms [48].

Improving the accuracy of the 3D breast outline segmentation may in turn improve the image quality near the skin line and potentially the visualization of skin thickening or retraction. Future work should also explore whether the visualization of invasive cancers behind the nipple is improved [49]. Ductal carcinoma in situ (DCIS) can be found or even masked in these areas [50]. For example, Paget’s disease [49,50] of the nipple is a rare condition associated with the extension of DCIS from the underlying ducts. Importantly, some erosive adenomatosis of the nipple can mask Paget’s disease [51] future work should investigate if improvements in the representation of the 3D breast outline and curvature facilitate superior detection and characterization of these rare breast cancers.

The detection and characterization of breast lesions can be improved with the reduction of out-of-plane artifacts, resulting in better image quality for the DBT exams. Our study described a method to improve the segmentation of breast tissue using T and XWR geometries. That said, computer simulations of pathological tissue (e.g., masses composed of soft tissue) must be performed to validate the benefits of the various imaging acquisitions. In future work, the impact of customized imaging acquisitions in the segmentation of breast lesions will be investigated.

The representation of the breast anatomy had some limitations. The PCA-based method can model the compressed breast curvature up to 85 mm; therefore, the distribution of CBT of the simulated population (56.10 [49.05, 66.65] mm) was not exactly matched (*p* < 0.001) to the clinical data (60.50 [51.50, 69.5] mm). No statistical differences between the means in the %VBD distributions were found (*p* = 0.17). In addition, complex mammary parenchyma should be simulated to represent both healthy and pathological nuances in breast anatomy. However, the simplified representation of breast tissues provided a better understanding of the X-ray physics in tomosynthesis imaging; this study presented evidence in support of the PA scanning motion unique to the NGT system, potentially improving the image quality for breast cancer screening and diagnosis. Future work should include the performance evaluation of nnU-Net and other AI architectures for the segmentation of complex breast tissue.

Ultimately, intelligent models that predict the convex hull and differentiate breast tissues will be incorporated into the NGT design to provide more reliable information about X-ray attenuation in the convex hull and adipose/dense tissue. The primary goal of the PET–DBT scanner is to monitor and potentially govern breast cancer treatment (i.e., diagnostic imaging). Our previous study demonstrated that improvements in tissue and breast outline segmentations may provide better co-registration of the breast anatomy (DBT) with radiotracer uptake (PET) [15,16]. The DBT component can ensure that breast lesion uptake measurements are less biased, while facilitating accurate breast delineation as well as providing information related to the breast tissue composition.

## 5. Conclusions

The proposed loss method could be used to train U-Net-based models to better segment the convex hull volume and, simultaneously, segment the volumes of dense and adipose tissues from DBT reconstructions. In addition, a geometry that incorporates the PA scanning motion (not used in the current clinical DBT systems) could improve the accuracy of the segmentation of the 3D breast outline and the segmentation of dense/adipose tissues, especially in the topmost and bottommost slices in DBT reconstructions.

Additional geometries will be further explored to maximize the benefits of next-generation scanning methods in our PET–DBT prototype. This VCT study provided evidence that next-generation scanning methods with PA motion improved breast tissue segmentation. Our results showed that the volumetric segmentation was either superior or comparable to an extra-wide-range geometry.

## Figures and Tables

**Table 1:** Patient demographics used to simulate virtual breast phantoms.

Demographics	
Patients, count	660
Age, years, mean ± SD	59.3 ± 10.7
BMI, mean ± SD	28.7 ± 7.7
Density BI-RADS (1, 2, 3, 4), count (%)	(79 (12), 367 (56), 201 (30), 13 (2))
Race (Asian, Black, White, Other), count	(25 (4), 309 (47), 320 (48), 6 (1))
CBT, mm, median (1st, 3rd) IQ	60.50 (51.50, 69.5)
%VBD, median (1st, 3rd) IQ	12.75 (8.40, 20.00)

**Table 2:** DBT geometry used to simulate and optimize acquisitions of the PET–DBT system.

Acquisition Parameters	(I) Conventional	(II) T	(III) XWR
X-ray Source Direction (Translation of focal spot)	Linear Unidirectional (LR)	Linear Bidirectional (LR + PA)	Angular Unidirectional (LR)
Number of Acquisitions (n)	15	15	91
Radiation Exposure (mode)	AEC	AEC	6 × AEC
Angular Range (°)	−7.5 to 7.5	−7.5 to 7.5	−45 to 45

**Table 3:** Summary of (A) Jaccard distance and (B) classification of background voxels categorized by *d* and models. Negative values of *d* represent the bottom portion of the phantom in the DBT reconstructions closer to the simulated breast support.

…	…	(A) Jaccard Distance (Model), Mean ± SD				(B) Improvement in Classification of Air				
d (mm)	Slices (#)	I_1_	I_1&2_	II_1&2_	III_1&2_	I_1&2_/I_1_	II_1&2_/I_1_	III_1&2_/I_1_	II_1&2_/I_1&2_	II_1&2_/III_1&2_
−37.30	2751	0.064 ± 0.037	0.044 ± 0.026	5.6 × 10^−4^ ± 9.7× 10^−4^	0.010 ± 0.006	31.95%	99.17%	84.27%	98.77%	94.70%
−22.40	2720	0.031 ± 0.023	0.027 ± 0.017	2.3 × 10^−4^ ± 5.6 × 10^−4^	0.005 ± 0.005	10.29%	99.34%	80.29%	99.26%	96.65%
−16.45	2725	0.015 ± 0.016	0.016 ± 0.012	8.3 × 10^−5^ ± 5.5 × 10^−4^	0.003 ± 0.002	−11.93%	99.54%	78.42%	99.60%	97.90%
−11.00	2750	0.007 ± 0.009	0.006 ± 0.007	1.9 × 10^−5^ ± 5.8 × 10^−5^	0.001 ± 0.001	−1.70%	99.72%	73.07%	99.73%	98.97%
−5.50	2725	0.003 ± 0.005	0.003 ± 0.004	1.4 × 10^−5^ ± 6.0 × 10^−5^	0.001 ± 0.001	20.86%	99.60%	61.80%	99.50%	98.97%
−0.05	2725	0.002 ± 0.003	0.001 ± 0.002	1.1 × 10^−5^ ± 5.6 × 10^−5^	6.8 × 10^−4^ ± 4.8 × 10^−4^	28.56%	99.39%	54.65%	99.15%	98.65%
5.40	2750	0.002 ± 0.007	0.001 ± 0.002	9.4 × 10^−5^ ± 3.8 × 10^−5^	3.9 × 10^−4^ ± 4.9 × 10^−4^	16.99%	99.28%	71.48%	99.13%	97.48%
10.90	2725	0.006 ± 0.019	0.002 ± 0.006	1.3 × 10^−5^ ± 4.3 × 10^−5^	4.3 × 10^−4^ ± 0.001	34.92%	99.62%	91.37%	99.42%	95.60%
16.35	2737	0.012 ± 0.033	0.004 ± 0.012	5.6 × 10^−5^ ± 2.5 × 10^−4^	9.3 × 10^−4^ ± 0.004	33.06%	99.52%	94.05%	99.29%	91.97%
22.30	2684	0.011 ± 0.009	0.007 ± 0.007	9.9 × 10^−5^ ± 3.4 × 10^−4^	4.9 × 10^−4^ ± 6.9 × 10^−4^	33.20%	99.10%	95.26%	98.65%	80.92%
Slice Average, 95% CI (low, high)		0.015 ± 0.027 (0.014, 0.015)	0.012 ± 0.019 (0.011, 0.012)	1.1 × 10^−4^ ± 4.1 × 10^−4^ (1.0 × 10^−4^, 1.1 × 10^−4^)	0.002 ± 0.004 (2.2 × 10^−3^, 2.3 × 10^−3^)	22.21% …	99.29% …	83.10% …	99.09% …	96.82% …

**Table 4:** Summary of Jaccard indices of (A) adipose and (B) dense tissue segmentation categorized by *d* and models. Negative values of *d* represent the bottom portion of the phantom in the DBT reconstructions closer to the simulated breast support.

…	…	(A) Jaccard of Adipose Tissue (Model), Mean ± SD				(B) Jaccard of Dense Tissue (Model), Mean ± SD			
d (mm)	Slices (#)	I_1_	I_1&2_	II_1&2_	III_1&2_	I_1_	I_1&2_	II_1&2_	III_1&2_
−37.30	2751	0.91 ± 0.06	0.92 ± 0.06	0.94 ± 0.06	0.98 ± 0.05	0.69 ± 0.22	0.72 ± 0.22	0.66 ± 0.22	0.90 ± 0.17
−22.40	2720	0.91 ± 0.08	0.91 ± 0.08	0.93 ± 0.06	0.97 ± 0.03	0.74 ± 0.13	0.76 ± 0.13	0.72 ± 0.16	0.92 ± 0.06
−16.45	2725	0.91 ± 0.06	0.92 ± 0.06	0.93 ± 0.05	0.98 ± 0.02	0.76 ± 0.10	0.78 ± 0.10	0.75 ± 0.10	0.94 ± 0.04
−11.00	2750	0.92 ± 0.06	0.92 ± 0.05	0.93 ± 0.05	0.98 ± 0.02	0.76 ± 0.09	0.78 ± 0.08	0.76 ± 0.08	0.94 ± 0.04
−5.50	2725	0.92 ± 0.05	0.92 ± 0.05	0.93 ± 0.05	0.98 ± 0.03	0.76 ± 0.08	0.77 ± 0.08	0.75 ± 0.08	0.93 ± 0.03
−0.05	2725	0.93 ± 0.04	0.93 ± 0.04	0.93 ± 0.04	0.98 ± 0.04	0.74 ± 0.10	0.77 ± 0.09	0.74 ± 0.10	0.93 ± 0.04
5.40	2750	0.93 ± 0.04	0.94 ± 0.04	0.94 ± 0.04	0.98 ± 0.03	0.75 ± 0.11	0.77 ± 0.10	0.74 ± 0.10	0.93 ± 0.04
10.90	2725	0.93 ± 0.06	0.94 ± 0.05	0.94 ± 0.05	0.98 ± 0.04	0.76 ± 0.11	0.78 ± 0.10	0.75 ± 0.11	0.94 ± 0.04
16.35	2737	0.92 ± 0.08	0.93 ± 0.07	0.93 ± 0.07	0.98 ± 0.03	0.73 ± 0.15	0.76 ± 0.13	0.71 ± 0.16	0.94 ± 0.04
22.30	2684	0.89 ± 0.07	0.90 ± 0.08	0.93 ± 0.08	0.97 ± 0.07	0.64 ± 0.24	0.67 ± 0.24	0.64 ± 0.23	0.86 ± 0.25
Slice Average 95% CI (low, high)		0.92 ±0.06 (0.915, 0.917)	0.92 ± 0.06 (0.923, 0.924)	0.93 ± 0.06 (0.931, 0.932)	0.98 ± 0.04 (0.977, 0.978)	0.73 ± 0.15 (0.731, 0.735)	0.76 ± 0.14 (0.755, 0.759)	0.72 ± 0.15 (0.719, 0.723)	0.92 ± 0.10 (0.921, 0.924)

**Table 5:** Summary of metrics collected for (A) adipose and (B) dense tissue segmentation.

…	(A) Adipose Tissue Segmentation (Model), Mean ± SD				(B) Dense Tissue Segmentation (Model), Mean ± SD			
Metric	I_1_	I_1&2_	II_1&2_	III_1&2_	I_1_	I_1&2_	II_1&2_	III_1&2_
Dice	0.91 ± 0.03	0.92 ± 0.03	0.93 ± 0.03	0.98 ± 0.02	0.75 ± 0.04	0.78 ± 0.03	0.74 ± 0.04	0.94 ± 0.01
Precision	0.95 ± 0.04	0.96 ± 0.04	0.96 ± 0.04	0.99 ± 0.01	0.86 ± 0.06	0.87 ± 0.05	0.85 ± 0.07	0.97 ± 0.01
Recall	0.96 ± 0.02	0.96 ± 0.02	0.97 ± 0.02	0.99 ± 0.02	0.89 ± 0.04	0.89 ± 0.04	0.86 ± 0.05	0.97 ± 0.02
Accuracy	0.99 ± 0.01	0.99 ± 0.01	0.99 ± 0.01	0.99 ± 0.00	0.99 ± 0.01	0.99 ± 0.01	0.99 ± 0.01	0.99 ± 0.00

## Data Availability

The data generated during the current study are available from the corresponding author on reasonable request.
